# Cardiovascular risk after cancer in patients with ischemic heart disease or peripheral artery disease

**DOI:** 10.1186/s40959-026-00520-z

**Published:** 2026-07-02

**Authors:** Torben Bjerregaard Larsen, Ricco Noel Hansen Flyckt, Margrethe Bang Henriksen, Claus Lohman Brasen, Torben Frøstrup Hansen, Flemming Skjøth

**Affiliations:** 1 Research Support Unit, Vejle Hospital, University Hospital of Southern Denmark, Beriderbakken 4, Vejle, DK-7100 Denmark; 2 Institute of Regional Health Research, University of Southern Denmark, Odense, Denmark; 3 Department of Oncology, Vejle Hospital, University Hospital of Southern Denmark, Vejle, Denmark; 4 Department of Biochemistry and Immunology, Vejle Hospital, University Hospital of Southern Denmark, Vejle, Denmark

**Keywords:** Cardio oncology, Atherosclerotic cardiovascular disease, Major adverse cardiovascular events, Venous thromboembolism, Population based cohort study, Secondary cardiovascular prevention, Risk prediction, Ischemic heart disease, Peripheral arterial disease, Cancer, Cardiovascular mortality

## Abstract

**Background:**

Patients with ischemic heart disease (IHD) or peripheral artery disease (PAD) represent a high-risk cardiovascular phenotype. When cancer is diagnosed in this population, clinical attention may shift toward treatment of the malignancy, potentially leading to under-recognition of pre-existing cardiovascular disease and its associated risks. Real-world data describing early cardiovascular outcomes after cancer diagnosis in this subgroup are however limited. The objective was to describe 1-year risks of cardiovascular death, major adverse cardiovascular events (MACE: ischemic stroke, systemic embolism, myocardial infarction, and hemorrhagic stroke), and venous thromboembolism (VTE: pulmonary embolism, and deep vein thromboembolism) after cancer diagnosis and to compare these risks across cancer sites within this high-risk vascular population.

**Methods:**

Using nationwide Danish registries, we identified 91,253 patients with IHD and/or PAD who developed a first primary cancer between 2002 and 2022. One-year cumulative incidences of cardiovascular death, MACE, and VTE were estimated using competing-risk methods. Multivariable Cox regression models were used to identify clinical predictors of cardiovascular outcomes.

**Results:**

Within one year of cancer diagnosis, 33% of patients died. The cumulative incidence of cardiovascular death was 2.4%, MACE 2.8%, and VTE 1.8%, with substantial variation across cancer types. Neurologic, respiratory, and digestive tract cancers were associated with the highest cardiovascular risk. Prior cardiovascular comorbidity strongly predicted adverse outcomes. Statin therapy was associated with lower cardiovascular mortality (hazard ratio [HR] 0.66) and fewer MACE events (HR 0.84).

**Conclusions:**

Cancer patients with pre-existing IHD or PAD experience a high burden of early cardiovascular and thrombotic events with marked variation across cancer types. These findings underscore the need for integrated cardio-oncology care and support continued use of guideline-directed cardiovascular preventive therapies during cancer treatment.

**Graphical Abstract:**

**Figure:**
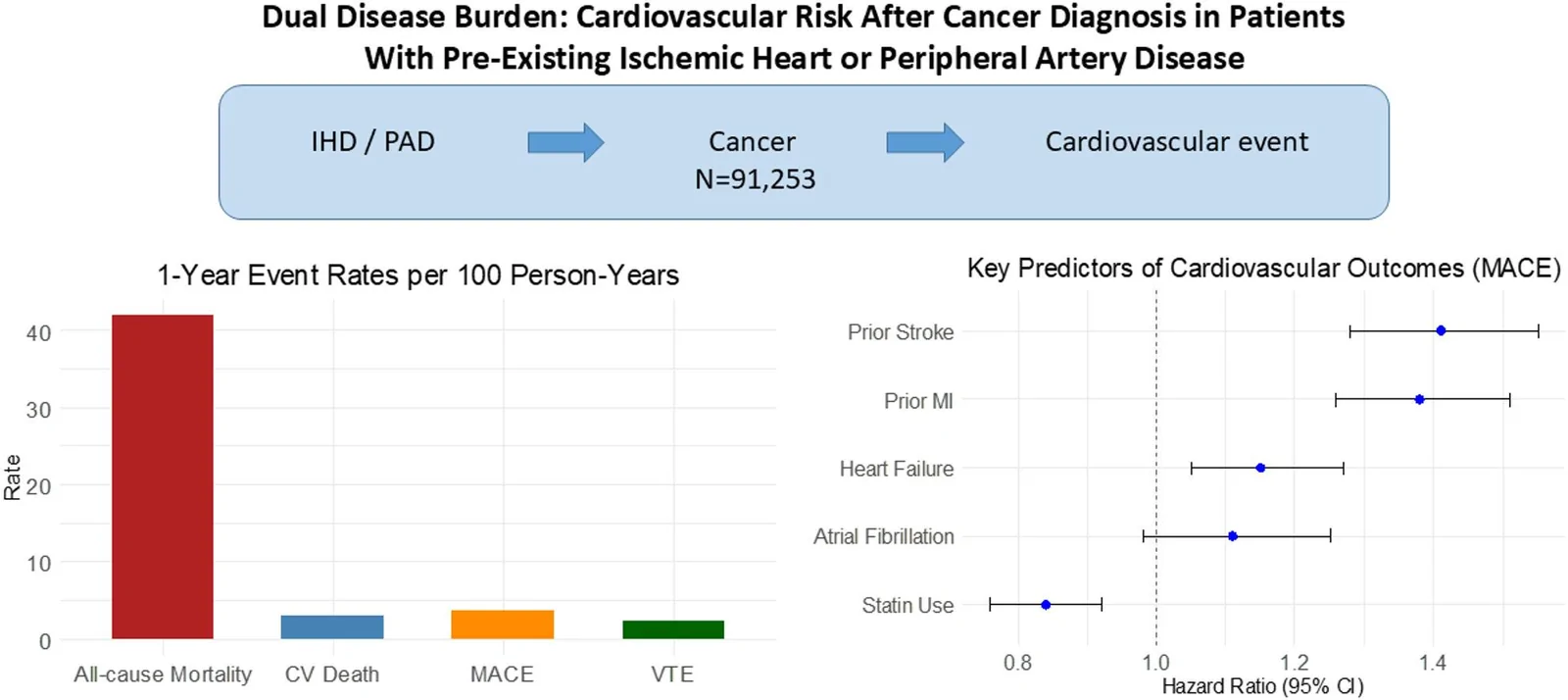

**Supplementary Information:**

The online version contains supplementary material available at https://doi.org/10.1186/s40959-026-00520-z.

## Introduction

Ischemic heart disease (IHD) and peripheral artery disease (PAD) are serious manifestations of systemic atherosclerosis and are associated with high rates of morbidity and mortality [1]. Many patients with IHD or PAD require intensive cardiovascular care, including anticoagulation, revascularization procedures, and commonly present with additional comorbidities such as diabetes, heart failure, and atrial fibrillation. Cancer is also prevalent in this population, driven by shared risk factors including older age, smoking, and chronic inflammation [2]. Patients with IHD or PAD represent an advanced vascular phenotype characterized by endothelial dysfunction, chronic inflammation, and heightened thrombotic potential. These biological features may be amplified during cancer development through systemic inflammatory activation, coagulation pathway stimulation, and cancer-associated metabolic stress [2–4]. Consequently, the vascular system is directly challenged when cancer arises in patients with existing atherosclerotic disease, but the magnitude and timing of early cardiovascular complications in this phenotype remain poorly quantified in population-based settings.

Although cancer and cardiovascular disease are typically managed along separate clinical pathways, their interaction is increasingly recognized. Cancer therapies can accelerate cardiovascular injury, while underlying vascular disease may limit treatment options or increase the risk of toxicity. Patients with IHD or PAD who develop cancer represent a particularly vulnerable subgroup, often excluded from clinical trials and underrepresented in current cardio-oncology guidelines [5].

Accurate cardiovascular risk stratification is critical in oncology to guide surveillance and inform treatment decisions. Previous studies have shown that risk stratification based on routinely collected registry data may be feasible and clinically informative. By describing short-term cardiovascular risk and their associations with clinical characteristics in cancer populations with pre-existing atherosclerotic disease a basis could be given for future work to help personalize and improve cardio-oncological care [6, 7].

Understanding vascular risk in this population is essential not only for clinical management but also for clarifying how cancer-related biological stressors interact with pre-existing atherosclerotic disease and thrombotic pathways. In this study, we used nationwide registry data to analyze a large population of Danish patients with IHD and/or PAD who subsequently developed cancer with the aim to describe one-year risks of all-cause mortality, cardiovascular death, major adverse cardiovascular events (MACE), and venous thromboembolism (VTE). We compared the risk of outcome across cancer sites and further examined clinical characteristics associated with these outcomes.

## Methods

### Data sources

This study used data from four nationwide Danish health registries, all linked at the individual level through a unique personal identification number assigned to every Danish resident at birth or upon immigration [8]. These registries offer comprehensive longitudinal data on demographics, comorbidities, healthcare utilization, and mortality.

The Danish National Patient Register, established in 1977, includes diagnostic codes and records of medical procedures from all somatic hospital admissions [9]. We classified diagnoses using the International Classification of Diseases, 10th revision (ICD-10), which has been implemented in Denmark since 1997. The Danish National Prescription Registry contains data on all redeemed prescriptions since 1994 and was used to identify pharmacologic exposures [10]. We categorized pharmacologic treatments according to the Anatomical Therapeutic Chemical (ATC) Classification System.

Demographic data—including date of birth, sex, migration status, and vital status—were obtained from the Danish Civil Registration System [8]. Information on cause-specific mortality was retrieved from the Danish National Cause of Death Register, which was available since 2002 [11].

Access to the data used in this study was granted through the Open Patient data Explorative Network (OPEN), Odense University Hospital, Region of Southern Denmark, and Statistics Denmark. The study was approved by the Danish Data Protection Agency via institutional registration with the Region of Southern Denmark (reference 23‑50300). Access to Danish health registry data requires affiliation with, or a formal collaboration involving, a Danish research institution as well as prior authorization from the relevant data custodians. All data are stored and analyzed within secure research environments administered by Statistics Denmark and cannot be transferred, downloaded, or exported for external use.

### Study population and outcomes

Cardiovascular outcomes were selected to reflect biologically linked processes involving atherosclerotic plaque instability (MACE), thrombotic events (VTE), and vascular mortality pathways.

We identified all patients since 1997 with a hospital discharge diagnosis of ischemic heart disease (IHD; ICD-10 codes I20–I25) or peripheral artery disease (PAD; ICD-10 codes I70–I74, I77), excluding subcodes representing other peripheral vascular conditions (I708A, I713, I714, I730, I731, I738, I739B). Patients were included if they subsequently received a hospital discharge diagnosis of cancer (ICD-10 codes C00–C97) between January 1, 2002, and June 30, 2022.

Patients with ICD-10 code C44 (“Other and unspecified malignant neoplasms of skin”) were excluded. This code captures nonmelanoma skin cancers, including basal cell carcinoma and squamous cell carcinoma, but excludes melanoma, which is classified under code C43.

The date of cancer diagnosis was defined as the index date for follow-up.

To ensure data quality and identify incident cases, patients less than 1 year of residence in Denmark prior to cancer diagnosis were excluded, as well as patients with inconsistent vital status information. Individuals with a history of cancer before 2002 or preceding the diagnosis of IHD or PAD were also excluded, as were those diagnosed with unspecified or metastatic cancers (ICD-10 codes C76–C80). To account for variation in cancer prognosis, malignancies were categorized by anatomic site: digestive tract (C00–C26); respiratory organs, including intrathoracic structures (ICD-10 codes C30–C39); bone, skin, and connective tissue (C40–C49); glandular and reproductive organs (C50–C63, C73–C75); uro-renal system (C64–C68); neurologic system (C69–C72); and hematologic malignancies (C81–C99). These groupings reflect common anatomic divisions and broadly similar treatment considerations within each category. In case of multiple sites, the patient was classified according to the site with first ICD-10 code in lexicographic order (C00-C97). Each individual was thus assigned to a single, mutually exclusive cancer site category.

Primary outcomes were MACE, VTE and cardiovascular death. Cardiovascular death was identified using the Danish Cause of Death Register. MACE was defined as ischemic stroke, systemic embolism, myocardial infarction, or hemorrhagic stroke. Only primary discharge diagnoses from acute hospital encounters were included to avoid misclassifying follow-up visits for prior cardiovascular events as incident events. VTE included pulmonary embolism and deep vein thrombosis. To improve diagnostic specificity, VTE events were included only if the diagnosis was recorded within 10 days of a procedure code indicating imaging or diagnostic evaluation for emboli in the lungs or lower extremities [12]. The outcome of all-cause death was included to evaluate overall mortality. To assess the sensitivity of the analyses to outcome definition, we also considered events identified from any type of hospital contact and from both primary and secondary diagnoses. For MACE and VTE, we repeated the analyses in the subgroup without prevalent outcome disease at baseline to focus on incident events only. Sensitivity to the number of days required to qualify a VTE diagnosis was evaluated by allowing up to 20 days between diagnosis and the corresponding diagnostic procedure. Diagnostic and procedural codes used to define each outcome are listed in Supplementary Table 1.

Baseline cardiovascular risk factors were assessed using routinely collected administrative data. At the index date, we recorded diagnoses of atrial fibrillation, ischemic stroke, VTE, major bleeding, myocardial infarction, kidney disease, liver disease, heart failure, diabetes mellitus, hypertension, rheumatic disease or systemic lupus erythematosus, and thyroid dysfunction (hypo- or hyperthyroidism). Diagnoses of hypertension and diabetes mellitus required evidence of pharmacologic treatment.

Patients were classified as receiving cardiovascular prophylaxis if they filled at least 1 prescription for a statin, antiplatelet agent, or oral anticoagulant in the year before their cancer diagnosis. Overall comorbidity burden was assessed using the Charlson Comorbidity Index, categorized as 0–3, 4, 5, 6, or 7–21 points [13]. To estimate stroke and cardiovascular risk, we calculated the CHA₂DS₂-VASc score [14]. The full list of codes and weights used to define risk factors is provided in Supplementary Table 1.

### Statistical analyses

Patients were characterized descriptively at baseline regarding distributions of sex, age, index vascular phenotypes (IHD and/or PAD), as well as clinical and medical covariates mentioned above, stratified by cancer site. Time-to-event methods were used to estimate the risk of outcomes and examine associations with cardiovascular risk factors. Time at risk was defined as the period from the cancer diagnosis date (index date) to the earliest of the following: outcome occurrence, death, emigration, 1 year of follow-up, or December 31, 2022.

Crude incidence rates were calculated as the number of events per person-years at risk, reported both overall and stratified by cancer site. To account for competing risks, cumulative incidence functions for MACE and VTE were estimated using the Aalen–Johansen estimator, with all-cause death treated as a competing event. For cardiovascular death, non-cardiovascular deaths were considered competing events. Given the high mortality in this population, we also reported all-cause mortality rates and estimated survival using the Kaplan–Meier method.

Cause specific associations between rate of outcomes and clinical characteristics were assessed using Cox proportional hazards regression considering non-cardiovascular death as a censoring event. Models included sex, cancer site (categorical), age (modeled as a continuous variable using cubic splines), Charlson Comorbidity Index (CCI) group, and the individual cardiovascular risk indicators defined above as exposures of interest. Both crude and adjusted hazard ratios (HRs) were reported. Adjusted estimates were obtained from multivariable models including all listed covariates, except CCI. The proportional hazards assumption was assessed using Schoenfeld residual plots, with no violations requiring alternative modelling identified. The adjusted effects of CCI group were adjusted only for predictors not included in the CCI. Given the expected high mortality, we included a supplementary Fine and Gray regression to assess whether the direction and relative magnitude of the associations were consistent with the observed differences in cumulative incidence. Non-cardiovascular death was treated as a competing risk in these analyses, which should be considered supportive only. In regression analyses comparing risks across cancer sites, bone, skin, and connective tissue cancer was used as the internal reference category, as this group had comparatively low short-term mortality and cardiovascular event rates within the cohort.

All data management and statistical analyses were performed using SAS version 9.4 (SAS Institute Inc), Stata/MP version 18.0 (StataCorp LLC), and R Statistical Software version 4.3.0 (R Foundation for Statistical Computing).

## Results

We initially identified 126,735 patients with IHD and/or PAD who were diagnosed with a first primary cancer between 2002 and 2022. After applying exclusion criteria based on registry data and timing of diagnoses, 91,253 patients were included in the final study population.

**Fig. 1:**
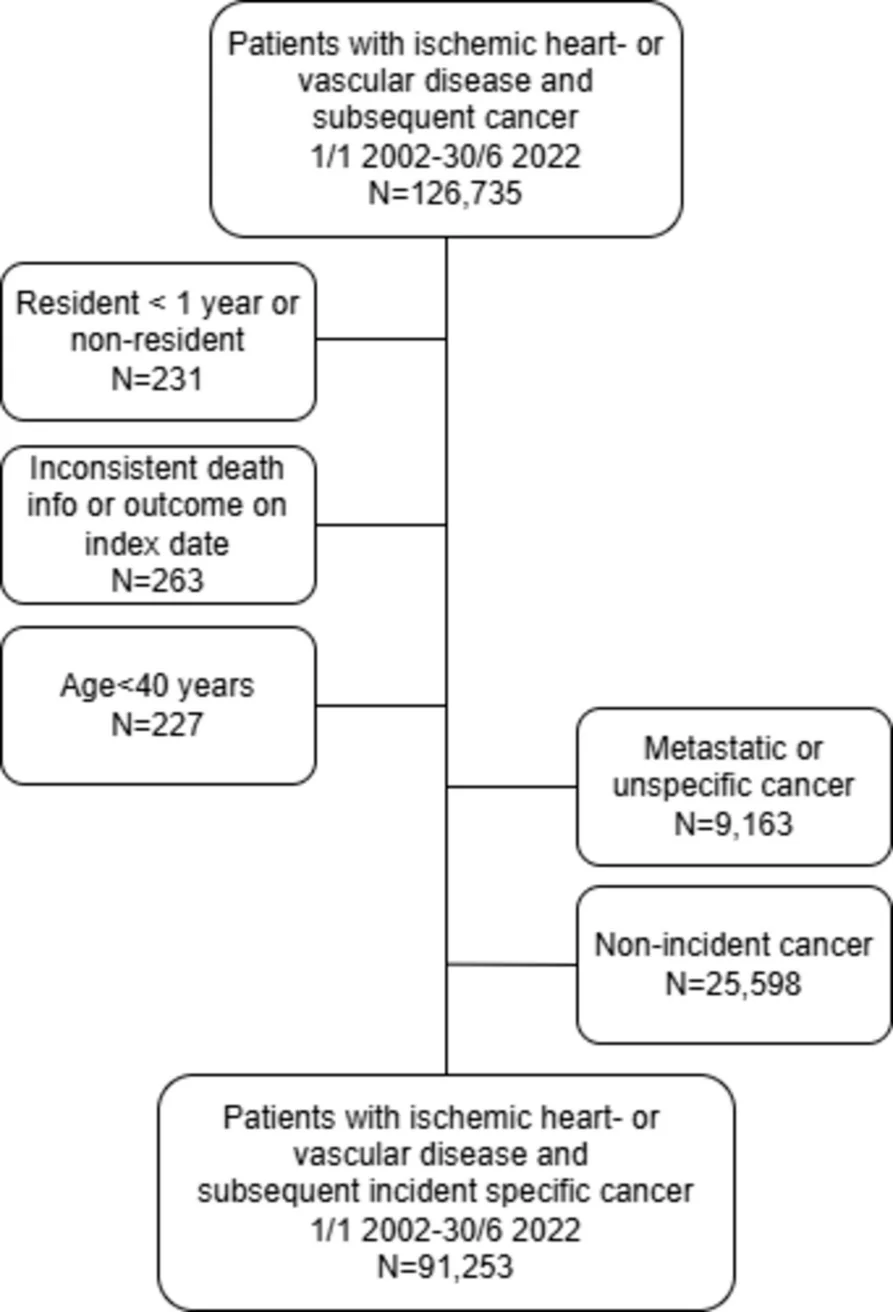
Inclusion of patients. PAD: Peripheral artery disease; VTE: Venous thromboembolism; CHA₂DS₂-VASc Score: congestive heart failure, hypertension, age ≥ 75 years (doubled), diabetes, stroke/transient ischemic attack/thromboembolism (doubled), vascular disease, age 65–74 years, female

The inclusion process is detailed in the study flowchart (Fig. 1), which outlines registry definitions, exclusion criteria, and cohort construction.

### Baseline characteristics

Baseline characteristics of the study population are shown in Table 1. The mean age was 73.7 years (SD, 9.6), and 36.7% were female. Ischemic heart disease was the primary index vascular phenotype in 79.3% of patients, 32.7% had PAD, and 12.0% had both conditions.

**Table 1:** Baseline demographics, cardiovascular comorbidities, and treatments by cancer type

Cancer location	Digestive tract	Respiratory	Bone, Skin, Tissue	Glandular, Reproductive	Uro-renal	Neurologic	Hematologic	All
N (% of total)	24,302 (26.6)	18,995 (20.8)	4,802 (5.3)	26,182 (28.7)	7,377 (8.1)	1,463 (1.6)	8,132 (8.9)	91,253 (100)
Female	34.3 (8,324)	39.3 (7,466)	33.7 (1,617)	42.2 (11,060)	23.4 (1,723)	36.7 (537)	33.7 (2,737)	36.7 (33,464)
Age mean (SD)	74.3 (9.7)	73.2 (8.6)	73.2 (10.8)	73.4 (9.6)	74.2 (9.4)	72.3 (10.2)	74.1 (9.9)	73.7 (9.6)
Age > 65	81.1 (19,700)	81.4 (15,467)	77.6 (3,727)	80.2 (20,993)	82.0 (6,050)	74.5 (1,090)	81.3 (6,609)	80.7 (73,636)
Ischemic heart disease	79.7 (19,375)	71.7 (13,626)	82.5 (3,963)	83.0 (21,728)	79.5 (5,868)	81.4 (1,191)	81.8 (6,651)	79.3 (72,402)
Peripheral artery disease	32.5 (7,902)	44.5 (8,448)	27.1 (1,299)	26.4 (6,906)	33.8 (2,491)	27.8 (406)	28.9 (2,354)	32.7 (29,806)
Prior stroke	19.4 (4,709)	20.8 (3,949)	17.6 (844)	17.0 (4,442)	19.8 (1,464)	26.5 (387)	17.7 (1,437)	18.9 (17,232)
Heart failure	38.3 (9,311)	37.9 (7,191)	32.7 (1,571)	33.7 (8,811)	38.7 (2,853)	31.6 (462)	37.7 (3,069)	36.5 (33,268)
Prior myocardial infarction	30.5 (7,409)	29.7 (5,635)	29.8 (1,429)	29.0 (7,605)	33.0 (2,436)	29.5 (432)	30.6 (2,486)	30.1 (27,432)
Atrial fibrillation	21.9 (5,325)	19.4 (3,679)	21.2 (1,019)	19.8 (5,179)	21.1 (1,553)	19.3 (282)	22.2 (1,802)	20.6 (18,839)
Venous thromboembolism	7.3 (1,779)	7.2 (1,373)	6.2 (300)	6.0 (1,563)	6.9 (506)	6.0 (88)	7.5 (613)	6.8 (6,222)
Diabetes mellitus	26.3 (6,395)	21.5 (4,083)	20.2 (972)	20.0 (5,229)	24.0 (1,769)	20.9 (306)	21.4 (1,742)	22.5 (20,496)
Hypertension	83.3 (20,245)	80.0 (15,200)	82.9 (3,979)	82.3 (21,558)	83.9 (6,190)	80.7 (1,180)	82.5 (6,706)	82.3 (75,058)
Statin treatment	61.8 (15,023)	64.9 (12,324)	62.5 (3,003)	62.0 (16,242)	65.8 (4,853)	62.2 (910)	60.4 (4,912)	62.8 (57,267)
Antiplatelet treatment	65.6 (15,946)	69.4 (13,178)	62.1 (2,984)	64.2 (16,809)	69.1 (5,099)	64.5 (943)	64.2 (5,222)	65.9 (60,181)
Oral anticoagulation	17.5 (4,257)	15.0 (2,847)	17.6 (843)	15.9 (4,170)	16.8 (1,240)	16.1 (236)	18.2 (1,480)	16.5 (15,073)
Charlson mean (SD)	4.2 (1.9)	4.3 (1.8)	3.8 (1.7)	3.8 (1.7)	4.2 (1.8)	4.0 (1.8)	4.2 (1.9)	4.1 (1.8)
CHA₂DS₂-VASc mean (SD)	4.1 (1.7)	4.2 (1.7)	3.8 (1.7)	3.9 (1.7)	4.1 (1.6)	4.0 (1.8)	4.0 (1.7)	4.0 (1.7)

Distribution of clinical characteristics, pharmacologic treatments, and risk scores among patients with ischemic heart disease and/or peripheral artery disease at the time of cancer diagnosis, stratified by cancer site. Venous thromboembolism includes pulmonary embolism and deep vein thrombosis

Respiratory: Respiratory and thoracic organs; CHA₂DS₂-VASc Score: congestive heart failure, hypertension, age ≥ 75 years (doubled), diabetes, stroke/transient ischemic attack/thromboembolism (doubled), vascular disease, age 65–74 years, female sex

Comorbidities were frequent, with a mean Charlson Comorbidity Index of 4.1 (SD, 1.8) and a mean CHA₂DS₂-VASc score of 4.0 (SD, 1.7). The most common comorbidities were hypertension (82.3%), diabetes mellitus (22.5%), heart failure (36.5%), atrial fibrillation (20.6%), and prior myocardial infarction (30.1%). Some comorbidities varied by cancer type: prior stroke was more prevalent among patients with neurologic cancers; PAD was more common in cancers of the respiratory system; and diabetes was more frequent in patients with digestive tract cancers.

Use of cardioprotective medications was high, with 62.8% receiving statins, 65.9% receiving antiplatelet therapy, and 16.5% treated with oral anticoagulants.

Cancer types were distributed as follows: glandular and reproductive organs (28.7%, primarily breast cancer in women and prostate cancer in men), digestive tract (26.6%), respiratory organs (20.8%, mainly lung cancer), hematologic malignancies (8.9%), uro-renal system (8.1%), bone, skin, and connective tissue (5.3%), and neurologic system (1.6%).

### Cardiovascular outcomes and death

Within 1 year of follow-up, 29,856 patients died, corresponding to an all-cause mortality rate of 42.0 per 100 person-years and a cumulative 1-year mortality of 32.9%. Mortality varied substantially across cancer types (Table 2, Supplementary Fig. 1). Patients with cancers of the glandular and reproductive organs had the lowest 1-year mortality (11.8%), whereas those with neurological cancers had the highest (61.8%).

**Table 2:** Cardiovascular outcomes and mortality by cancer type within 1 year of follow-up

	**Mortality**			**Cardiovascular death**		
**Cancer strata**	**Events**	**Event Rate** **pr 100 PY (CI)**	**1-year cumulative incidence (%) (CI)**	**Events**	**Event Rate** **pr 100 PY (CI)**	**1-year cumulative incidence (%) (CI)**
All	29,856	42.0 (41.5–42.5)	32.9 (32.5–33.2)	2,200	3.09 (2.97–3.23)	2.4 (2.3–2.5)
Digestive tract	9,875	56.4 (55.3–57.6)	40.8 (40.2–41.4)	655	3.74 (3.47–4.04)	2.7 (2.5–2.9)
Respiratory	10,697	91.3 (89.6–93.0)	56.6 (55.9–57.3)	414	3.53 (3.21–3.89)	2.2 (2.0–2.4)
Bone, Skin, Tissue	772	17.9 (16.7–19.2)	16.2 (15.2–17.3)	86	2.00 (1.61–2.46)	1.8 (1.5–2.2)
Glandular, reproductive	3,076	12.7 (12.3–13.2)	11.8 (11.4–12.2)	526	2.17 (1.99–2.37)	2.0 (1.9–2.2)
Uro-renal	2,213	37.0 (35.5–38.6)	30.2 (29.1–31.2)	228	3.81 (3.35–4.34)	3.1 (2.7–3.5)
Neurologic	900	102.9 (96.4–109.9)	61.8 (59.3–64.3)	33	3.77 (2.68–5.31)	2.3 (1.6–3.1)
Hematologic	2,323	35.9 (34.4–37.4)	28.7 (27.7–29.7)	258	3.98 (3.53–4.50)	3.2 (2.8–3.6)
	**MACE**			**VTE**		
All	2,517	3.59 (3.45–3.73)	2.8 (2.7–2.9)	1,658	2.36 (2.25–2.47)	1.8 (1.7–1.9)
Digestive tract	681	3.96 (3.67–4.26)	2.8 (2.6–3.0)	551	3.19 (2.94–3.47)	2.3 (2.1–2.5)
Respiratory	556	4.82 (4.44–5.24)	2.9 (2.7–3.2)	449	3.88 (3.54–4.26)	2.4 (2.2–2.6)
Bone, Skin, Tissue	100	2.34 (1.92–2.85)	2.1 (1.7–2.5)	40	0.93 (0.68–1.27)	0.8 (0.6–1.1)
Glandular, reproductive	648	2.71 (2.51–2.92)	2.5 (2.3–2.7)	304	1.26 (1.13–1.41)	1.2 (1.0–1.3)
Uro-renal	245	4.18 (3.69–4.73)	3.3 (2.9–3.8)	121	2.04 (1.71–2.44)	1.6 (1.4–2.0)
Neurologic	35	4.07 (2.92–5.67)	2.4 (1.7–3.3)	45	5.26 (3.93–7.05)	3.1 (2.3–4.1)
Hematologic	252	3.95 (3.49–4.47)	3.1 (2.8–3.5)	148	2.31 (1.97–2.72)	1.8 (1.6–2.1)

MACE includes ischemic stroke, systemic embolism, myocardial infarction, and hemorrhagic stroke. VTE includes pulmonary embolism and deep vein thrombosis, confirmed by diagnostic evaluation (imaging or ultra-sound). Only acute, primary diagnoses were included. Cardiovascular death was identified using the Danish Cause of Death Register. Incidences are reported as cumulative 1-year risks stratified by cancer site

*PY* per year, *CI* Confidence interval (95%)

Cardiovascular events occurred at lower rates (Table 2). A total of 2,200 patients died from cardiovascular causes (3.1 per 100 person-years), 2,517 experienced a major adverse cardiovascular event (MACE; 3.6 per 100 person-years), and 1,658 experienced a VTE event (2.4 per 100 person-years). The corresponding 1-year cumulative incidences were 2.4% for cardiovascular death, 2.8% for MACE, and 1.8% for VTE.

**Fig. 2:**
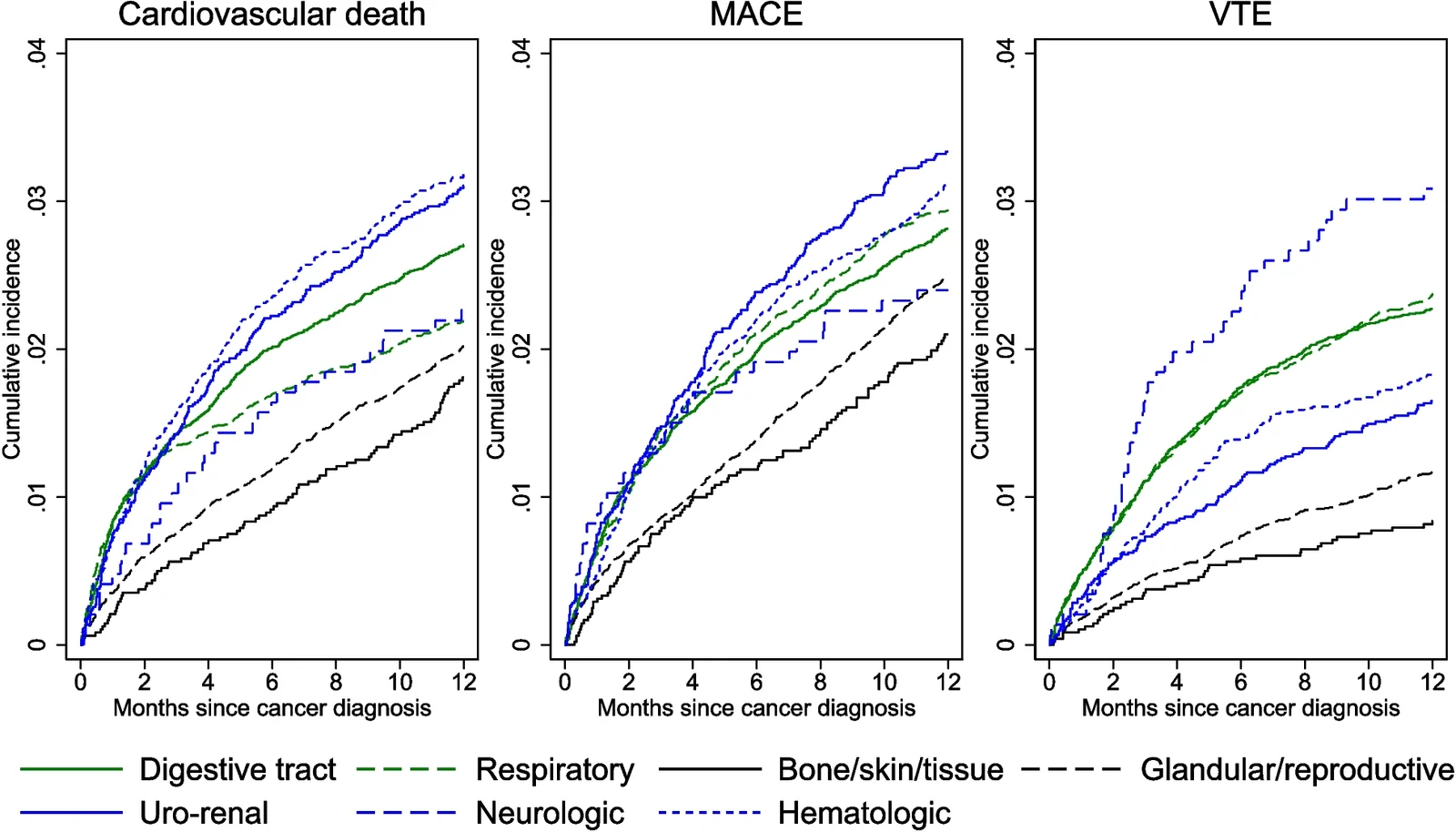
Cumulative 1-year incidence of cardiovascular events and death by cancer type. Curves represent cumulative incidence of cardiovascular death, MACE, and VTE over 12 months following cancer diagnosis, stratified by cancer site. MACE includes ischemic stroke, systemic embolism, myocardial infarction, and hemorrhagic stroke. VTE includes pulmonary embolism and deep vein thrombosis, confirmed by diagnostic evaluation (imaging or ultra-sound). Only acute, primary diagnoses were included. Cardiovascular death was identified using the Danish Cause of Death Register. Incidences are reported as cumulative 1-year risks stratified by cancer site

Figure 2 and Table 2 display cumulative incidence curves over the first year following cancer diagnosis, illustrating the timing and burden of cardiovascular events and death. The incidence of MACE and VTE varied by cancer type. MACE was most common among patients with uro-renal cancers (3.3%), and VTE incidence was highest among those with neurologic cancers (3.1%). In contrast, patients with bone, skin, and connective tissue cancers had the lowest incidence of both MACE (2.1%) and VTE (0.8%). Cardiovascular death was most frequent in patients with hematologic (3.2%) and uro-renal (3.1%) malignancies. Sensitivity analyses restricting the study population to subgroups without MACE or VTE at baseline slightly lowered the observed event rates and risks, but preserved the ordering across cancer groups (Supplementary Tables 2 and 3). Similar results were observed when allowing a longer interval for diagnostic confirmation of VTE (Supplementary Table 3). When including any primary or secondary codes from acute or elective outcome contacts, the number of MACE and VTE events increased by 81% to 4,557 and by 47% to 2,432, respectively. The ordering of outcome risk across cancer groups was maintained compared with the main analysis, except for MACE events in neurological cancer, which increased markedly.

### Predictors of cardiovascular outcomes

**Fig. 3:**
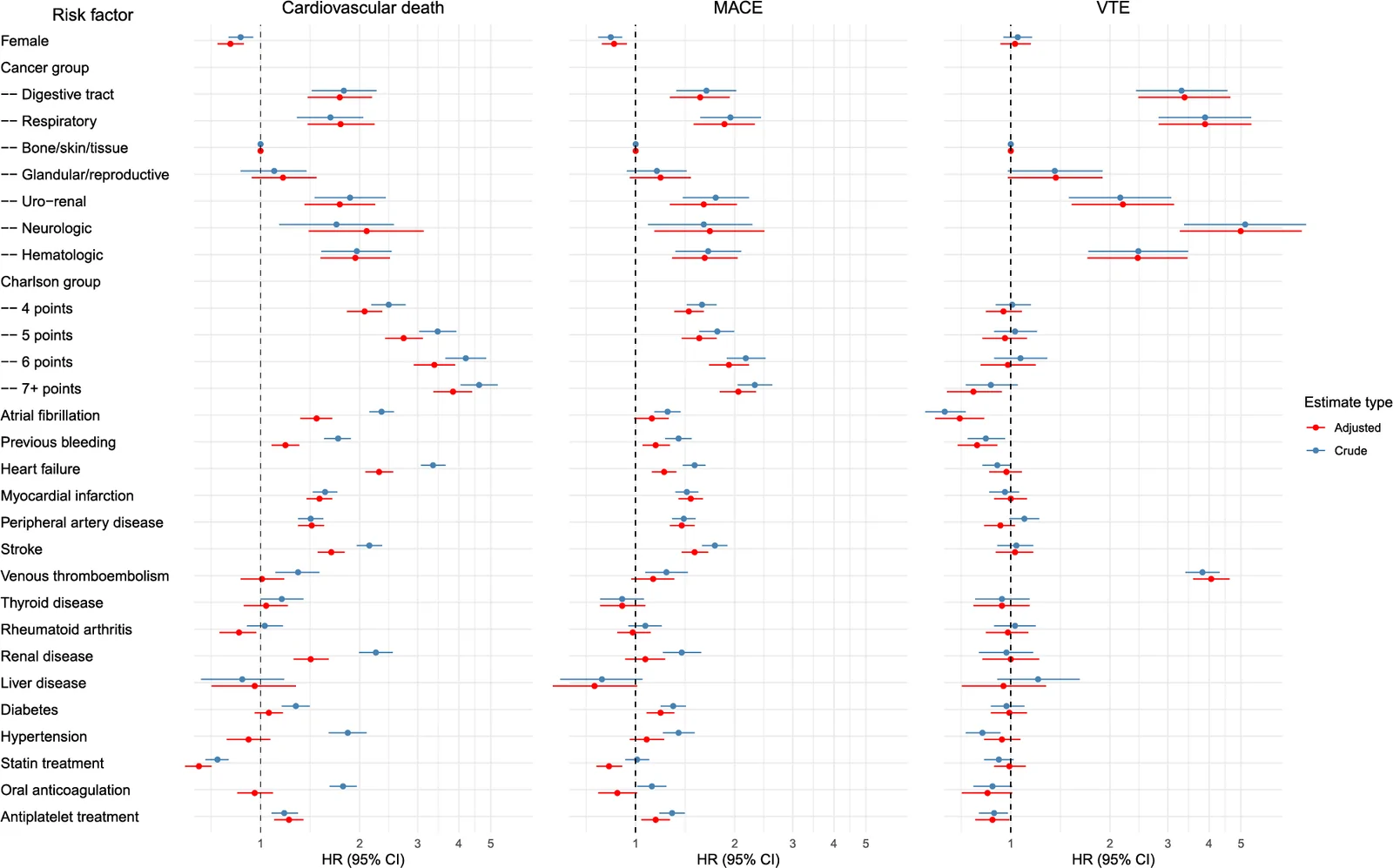
Risk factors for cardiovascular death, MACE and VTE after a cancer diagnosis. Forest plots of hazard ratios (HRs) and 95% confidence intervals forcardiovascular death, MACE, and VTE. Both crude (blue) and adjusted (red) estimates are shown for each risk factor. MACE includes ischemic stroke, systemicembolism, myocardial infarction, and hemorrhagic stroke. VTE includes pulmonary embolism and deep vein thrombosis, confirmed by diagnosticAQ10 evaluation (imaging or ultra-sound). Only acute, primary diagnoses were included. Exact numbers are reported in Supplementary Table 2

Crude and adjusted HRs for cardiovascular death, MACE, and VTE were evaluated across a wide range of clinical variables and cancer sites. A visual summary of key associations is presented in Fig. 3; full estimates are provided in Supplementary Table 4.

The rates of cardiovascular events were consistently elevated in the presence of comorbid conditions and varied by cancer type. Using bone, skin, and connective tissue cancers as the internal reference category, most other cancer sites had higher cause-specific hazards of cardiovascular events. This was consistent with their generally higher short-term mortality and cardiovascular burden in this IHD/PAD population. Most cardiovascular comorbidities present at the time of cancer diagnosis were associated with increased risk of both cardiovascular death and MACE. Notably, prior stroke (adjusted HR, 1.51; 95% CI, 1.38–1.66) and prior myocardial infarction (HR, 1.47; 95% CI, 1.35–1.60) were the most strongly associated with MACE.

Statin use was associated with significantly lower rates of cardiovascular death (HR, 0.65; 95% CI, 0.59–0.71) and MACE (HR, 0.83; 95% CI, 0.76–0.91). Similarly, recent use of oral anticoagulants (OAC) showed a tendency toward lower rates for both MACE and VTE. For VTE specifically, atrial fibrillation was associated with a lower rate (HR, 0.70; 95% CI, 0.59–0.83), and antiplatelet treatment were associated with lower VTE; whereas previous VTE as highly associated with a recurrent VTE diagnosis (HR, 4.06 (3.58–4.62)).

Cancer type remained a strong and independent determinant of cardiovascular outcomes. Compared with cancers of bone, skin, and connective tissue (reference group), all other cancer types, except for glandular and reproductive cancers, were associated with substantially higher risks of cardiovascular death and MACE. A similar pattern was observed for VTE, though effect sizes were generally larger. These conclusions were consistent when assessing the association between risk factors and the observed incidence of events, with non-cardiovascular death treated as a competing risk (Supplementary Table 5 and Supplementary Fig. 2).

These findings highlight the prognostic importance of both baseline comorbidity burden and preventive pharmacotherapy, particularly statins and OAC, as being associated with cardiovascular risk after a cancer diagnosis.

## Discussion

In this cohort of patients with pre-existing IHD and/or PAD who developed cancer, we observed high mortality and frequent cardiovascular and thrombotic events during the first year after cancer diagnosis. The variation observed across cancer groups may reflect differences in patient characteristics, cancer biology, treatment exposures, or a combination of these factors. However, causal mechanisms cannot be inferred from these data. The findings from this study highlight that early cardiovascular complications after cancer are not incidental but may reflect interactions between malignancy-related inflammation and pre-existing vascular disease. Patients with IHD or PAD carry a biologically primed vascular environment characterized by endothelial dysfunction, platelet activation, and coagulation pathway upregulation, increasing susceptibility to adverse events.

Although cardiovascular complications are increasingly recognized in cancer care, patients with established vascular disease remain underrepresented in cardio-oncology research and clinical guidelines. In this nationwide cohort of more than 91,000 patients with IHD and/or PAD newly diagnosed with cancer, we found a substantial burden of early mortality and cardiovascular events. Within the first year following cancer diagnosis, nearly one-third of patients died; nearly 3 percent experienced a MACE, and approximately 2 percent developed VTE.

The rate of cardiovascular death reached 3 per 100 person-years, underscoring the vulnerability of this dual-disease population. In these patients, systemic atherosclerosis may be further exacerbated by physiological and treatment-related stressors during cancer care.

Marked heterogeneity between cancer groups was observed. This variation is consistent with known differences in tumor-associated inflammation, coagulation pathway activation, metabolic stress, and treatment exposures described in prior literature. These factors may help explain the cardiovascular risk patterns seen across malignancies, although the underlying mechanisms cannot be confirmed within the present dataset. The comparatively higher VTE risk in neurologic cancers and the greater MACE burden in uro-renal cancers may reflect differing vascular risk profiles across malignancies, but these patterns should be interpreted cautiously given the observational design.

### Implications for vascular risk assessment

Cardiovascular risk varied substantially across cancer types. Neurologic, respiratory, and digestive tract cancers were associated with the highest one-year rates of cardiovascular death (Fig. 2). Notably, cardiovascular events and cancer-related death often occurred in close temporal proximity, complicating treatment planning and follow-up. Importantly, elevated cardiovascular risk was observed across all cancer groups, extending beyond traditionally recognized “cardiotoxic” malignancies.

In multivariable analyses (Fig. 3), several clinical predictors of adverse cardiovascular outcomes emerged consistently. Statin therapy was associated with reduced risk of cardiovascular death (adjusted HR, 0.65), MACE (HR, 0.83). The association with VTE risk was neutral (HR, 0.99). This is a clinically relevant finding but should be interpreted with caution given the wide range of sources for potential confounding factors. This association is biologically plausible given statins’ known effects on plaque stabilization, endothelial function, and systemic inflammation. However, because of the observational design and exposure definition, based on at least one prescription fill in the preceding year, these findings should not be interpreted as evidence of a protective causal effect. Residual confounding by frailty, healthcare engagement, indication, and other unmeasured factors, as well as healthy-user bias, are likely. The associations should therefore be considered hypothesis-generating and supportive of guideline-directed continuation of statin therapy, rather than proof that initiating or re-initiating statins at cancer diagnosis reduces event risk. In contrast, atrial fibrillation, heart failure, stroke, PAD, and higher comorbidity burden were associated with increased cardiovascular risk.

These findings align with prior work emphasizing the prognostic significance of cardiovascular health in oncology [15, 16]. However, most existing studies have focused on therapy-related cardiovascular injury rather than the impact of pre-existing cardiovascular conditions in cancer populations [17, 18].

### Implications for future risk stratification

While our study used traditional time-to-event analyses and did not aim to develop a formal prediction model, the observed absolute event rates and consistent associations with prior cardiovascular comorbidity suggest that registry-based risk stratification in this population is feasible. Future studies could build on these findings by developing and validating dedicated short-term cardiovascular risk models for patients with IHD/PAD at the time of cancer diagnosis, including assessments of calibration, discrimination, and clinical utility. Although large by cardio-oncology standards, our cohort remains too small—and the available data too limited—to support development of a modern, generalizable prediction model. Current guidelines emphasize the need for sufficient event counts, clearly defined clinical use cases, rigorous validation, and transparent reporting [19]. Even if more detailed data such as biomarkers or imaging parameters were available, many of these inputs would not be accessible at the bedside, limiting practical utility. For a risk tool to be implemented in routine care, it must be automatically populated with information readily available to clinicians.

Pooling all cancer types increases heterogeneity in baseline cardiovascular risk and likely limits model discrimination. Prior studies have shown that models based solely on routinely collected data offer limited predictive performance in oncology populations [20, 21]. While registry-based approaches may support broad population-level stratification, accurate prediction at the individual level will require incorporation of more detailed clinical information. A more feasible next step may be to focus on a single malignancy with shared cardiovascular risk mechanisms—lung cancer is an obvious candidate—and incorporate cancer-specific predictors into risk models.

Recent methodological reviews have cautioned against reliance on simplified sample-size rules and instead recommend formal sample-size calculations, careful modeling of continuous variables, and structured bias assessment, such as PROBAST, to reduce the risk of model failure [22].

Finally, prognostic models tend to be most reliable when outcomes are relatively infrequent. In high-prevalence settings such as ours, with 30 percent one-year mortality, miscalibration may lead to clinically meaningful misclassification, and predictive values are mathematically constrained. The limited predictive performance of registry-based models underscores that vascular risk in cancer patients is driven by dynamic biological processes, including inflammation, endothelial activation, and hypercoagulability—that are not captured by static clinical variables alone. Before expanding datasets, researchers should clarify the intended clinical use case and determine whether broad risk categorization, rather than precise individualized prediction, may be the more practical strategy for improving patient care.

Beyond shared risk factors and cancer-related inflammation, treatment-related cardiotoxicity and prothrombotic effects likely contribute to the early cardiovascular burden observed in our cohort. Several commonly used systemic therapies are known to increase the risk of left ventricular dysfunction and heart failure (e.g. anthracyclines, HER2-targeted agents, selected tyrosine kinase inhibitors), ischemic events (e.g. fluoropyrimidines, VEGF-pathway inhibitors), and arrhythmias or conduction disturbances [1–5]. In addition, radiotherapy to the thoracic region may accelerate coronary and valvular disease and promote pericardial pathology, particularly in patients with pre-existing atherosclerosis.

Modern cancer immunotherapies introduce further complexity. Immune checkpoint inhibitors have been linked to myocarditis, pericardial disease, arrhythmias, and accelerated atherosclerosis, whereas other targeted agents can induce hypertension, endothelial dysfunction, and microvascular injury, which may in turn precipitate MACE and VTE in a susceptible vascular phenotype [3–5]. Supportive treatments, including transfusions, erythropoiesis-stimulating agents, and high-dose corticosteroids, may additionally modulate thrombotic risk and hemodynamic load.

The heterogeneous pattern of cardiovascular events across cancer sites in our study is compatible with differences in typical treatment regimens, radiotherapy fields, and treatment intensity; however, we did not have systematic information on specific anticancer agents, doses, or radiotherapy exposure. Our results should therefore be interpreted as reflecting the *combined* impact of underlying cardiovascular disease, the cancer process itself, and contemporary cancer treatments, rather than permitting attribution of risk to any single mechanism.

### Strengths and limitations

To our knowledge, this is the first study to quantify cardiovascular morbidity and mortality in a nationwide real-world cohort of patients with IHD and/or PAD following cancer diagnosis using competing-risk methods and multivariable models. Our findings provide a strong rationale for more targeted research and the development of risk tools tailored to this vulnerable subgroup, which remains underrepresented in trials and underserved in routine care. These results may inform future clinical guidelines and support a shift toward more integrated cardiovascular care in oncology.

Several limitations should be acknowledged. We lacked information on cancer stage, specific oncologic treatments, and relevant biomarkers, all of which may influence cardiovascular risk. As a result, we cannot determine the extent to which the observed variation in cardiovascular event rates across cancer sites reflects differences in underlying tumor biology and prognosis, rather than differences in cardiotoxic treatment patterns. The observed associations should therefore be interpreted as hypothesis-generating with regard to treatment-related mechanisms. The use of administrative codes may have introduced misclassification, although this is likely non-differential. Furthermore, exclusion of metastatic and unspecified cancers may have led to underestimation of cardiovascular risk in advanced disease. Finally, the associations should be interpreted cautiously because of the observational nature of the data. Residual confounding cannot be excluded and may substantially bias the estimated associations. The results may reflect differences in frailty and comorbidity, as well as socioeconomic and educational factors that can influence the extent of healthcare contact.

Our study included only patients with IHD and/or PAD who subsequently developed cancer. It did not include a non-cancer IHD/PAD comparator group or a cancer cohort without prior atherosclerotic disease. Accordingly, our estimates should be interpreted as *within-cohort event rates and relative differences between cancer sites*, rather than as excess risk attributable to cancer or as a general baseline cardiovascular risk for broader oncology populations. Future studies linking this cohort to appropriately matched non-cancer IHD/PAD patients, or to cancer patients without pre-existing atherosclerotic disease, would be needed to quantify the absolute excess risk attributable to cancer.

Future research should bridge population-based vascular epidemiology with mechanistic studies to clarify how cancer perturbs atherosclerotic and thrombotic pathways. Understanding these interactions will be essential for developing targeted vascular interventions during cancer care.

## Conclusions

Patients with pre-existing ischemic heart disease or peripheral artery disease experience a substantial burden of early cardiovascular and thrombotic events following cancer diagnosis. These findings highlight the importance of integrated cardio-oncology care and support continued use of guideline-directed cardiovascular preventive therapies, including statins, during cancer treatment.

## Supplementary Information

Supplementary Material 1.

## Data Availability

The data used in this study are not publicly available due to Danish data protection regulations. Access to individual-level data requires approval from Statistics Denmark or the Danish Health Data Authority and affiliation with an approved research institution. Data can only be accessed through secure research environments.

## Abbreviations

IHD: Ischemic heart disease
PAD: Peripheral artery disease
MACE: Major adverse cardiovascular events
VTE: Venous thromboembolism
ICD-10: International Classification of Diseases, 10th Revision
ATC: Anatomical Therapeutic Chemical
HR: Hazard ratio
CI: Confidence interval
SD: Standard deviation
CHA₂DS₂ VASc: Congestive heart failure, hypertension, age ≥ 75 years (doubled), diabetes, stroke/TIA/thromboembolism (doubled), vascular disease, age 65–74 years, sex category (female)
