# Supplementary material for: Cardiovascular risk after cancer in patients with ischemic heart disease or peripheral artery disease
Source: Cardiooncology. 2026 Jul 2;12:117. doi: 10.1186/s40959-026-00520-z (PMC13595647; doi:10.1186/s40959-026-00520-z)
Supplement: Supplementary file 1 [file 40959_2026_520_MOESM1_ESM.docx]

**Cardiovascular Risk After Cancer in Patients with Ischemic Heart Disease or Peripheral Artery Disease**

**Supplemental Appendix**

Torben Bjerregaard Larsen MD, PhD, FESC (1,2), Margrethe Bang Henriksen MD, PhD (2,3) Ricco Noel Hansen Flyckt MSc (3), Claus Lohman Brasen MD PhD (2,4), Torben Frøstrup Hansen MD, PhD (2,3), Flemming Skjøth MSc, PhD (1,2).

1. Research Support Unit, Vejle Hospital, University Hospital of Southern Denmark

2. Institute of Regional Health Research, University of Southern Denmark, Odense, Denmark

3. Department of Oncology, Vejle Hospital, University Hospital of Southern Denmark

4. Department of Biochemistry and Immunology, Vejle Hospital, University Hospital of Southern Denmark

**Supplementary Table 1. ICD-10 and ATC codes for definitions of indicators**

| **Indicator** | **Diagnoses identified in hospital discharge registers by ICD-10 codes** | **Drugs identified in pharmacy prescription registers by ATC codes** |
| --- | --- | --- |
| Cancer group: - Aero - Bone, Skin, Tissue - Glandular, reproductive - Digestive tract - Hematologic - Neurologic - Uro-renal | C30-C39  C40-C43, C45-C49  C50-C63, C73-C75  C00-C26  C81-C99  C69-C72  C64-C68 |  |
| Major adverse cardiovascular event (MACE):  Stroke:  - Ischemic stroke  - Hemorrhagic stroke  Systemic embolism  Myocardial infarction | I63 I64 I60 I62  I74  I21 I23 |  |
| Venous thromboembolism (VTE):* - Pulmonary embolism - Deep Venous thromboembolism | I26 I801 I802 I803 I808 I809 I819 I636 I676 I822 I823 I828 I829 |  |
| Heart failure | I110 I130 I132 I420 I50 |  |
| Atrial Fibrillation | I48 |  |
| Peripheral vascular/ischemic disease (PAD) | I70 I71 I72 I73 I74 I77 excluding: I708A I713 I714 I730 I731 I738 I739B |  |
| Ischemic Heart Disease | I20 I21 I23 I24 I25 |  |
| Diabetes | E10 E11 E12 E13 E14 H360 O240 O241 O242 O243 | A10 |
| Hypertension | I10 I11 I12 I13 I15 | C02A C02B C02C C02DA C02L C03A C03B C03D C03EA C03X C07C C07D C08G C09BA C09DA C09XA52 C02DB C02DD C02DG C04 C05 C07 C07F C08 C09BB C09DB C09  C09BB04 C09DA C09DB C09CX01 C09DX04 C07B |
| Statins |  | C10 |
| Antiplatelets treatment: - aspirin  - clopidogrel  - prasugrel |  | B01AC06  B01AC04  B01AC22 |
| Oral anticoagulants:  - warfarin  - dabigatran - apixaban - rivaroxaban |  | B01AA03 B01AE07 B01AF02 B01AF01 |
| Bleeding | I850 I864A K226 K228F K250 K252 K254 K256 K260 K262 K264 K266 K270 K272 K274 K276 K280 K282 K284 K286 K290 K298A K625 K638B K638C K661 K838F K868G K920 K921 K922 I60 I61 I62 I690 I691 I692 E078B E274B G951A I312 I319A I230 J942 M250 R04 S259A S368A S368B S368D T143C T144A D500 D62 D683 D698 D699 R58 T792A T792B H052A H313 H356 H431 H450 |  |
| Thyriod disease:  - Hypothyrodism  - Hyperthyrodism | E00 E01 E02 E03 E04 E05 E06 |  |
| Rheumatoid arthritis: - Systemic Lupus - Rheumatic disorder | L93 M32 M00 M01 M02 M03 M05 M06 M07 M08 M09 M10 M11 M12 M13 M14 M46 M47 |  |
| Renal disease | E102 E112 E142 I120 I131 I132 I150 I151 N03N05 N07 N08 N110 N14 N15 N16 N18 N19 N26 N280 N391 Q61 |  |
| Liver disease | B150 B160 B162 B18 B190 I85 K700 K701 K702 K703 K704 K709 K71 K72 K73 K74 K760 K766 |  |
| Chronic Pulmonary Disease | J40 J41 J42 J43 J44 J45 J46 J47 J60 J61 J62 J63 J64 J65 J66 J67 J684 J701 J703 J841 J920 J961 J982 J983 |  |

* Diagnoses confirmed by hospital procedure codes (SKS-codes) for phlebography UXAG05, ventilation-perfusion examination WLHGS, angiography UXAG, UXAC10, MR venography UXZ52, CT-scan UXCG UXCC, ultra-sound of lower extremities UXUG

| **Charlson indicator** | **ICD-10** | **Weight** |
| --- | --- | --- |
| Myocardial infarction | I21 I22 I23 | 1 |
| Congestive heart failure | I50 I110 I130 I132 | 1 |
| Peripheral vascular disease | I70 I71 I72 I73 I74 I77 | 1 |
| Cerebrovascular disease | I60 I61 I62 I63 I64 I65 I66 I67 I68 I69 G45 G46 | 1 |
| Dementia | F00 F01 F02 F03 F051 G30 | 1 |
| Chronic Pulmonary disease | J40 J41 J42 J43 J44 J45 J46 J47 J60 J61 J62 J63 J64 J65 J66 J67 J684 J701 J703 J841 J920 J961 J982 J983 | 1 |
| Connective tissue disease | M05 M06 M08 M09 M30 M31 M32 M33 M34 M35 M36 D86 | 1 |
| Ulcer disease | K221 K25 K26 K27 K28 | 1 |
| Mild liver disease | B18 K700 K701 K702 K703 K709 K71 K73 K74 K760 | 1 |
| Diabetes Mellitus | E100 E101 E109 E110 E111 E119 | 1 |
| Hemiplegia | G81 G82 | 2 |
| Moderate/severe renal disease | I12 I13 N00 N01 N02 N03 N04 N05 N07 N11 N14 N17 N18 N19 Q61 | 2 |
| Diabetes Mellitus with chronic complications | E102 E103 E104 E105 E106 E107 E108 E112 E113 E114 E115 E116 E117 E118 | 2 |
| Any tumor | C0 C1 C2 C3 C4 C5 C6 C70 C71 C72 C73 C74 C75 | 2 |
| Leukemia | C91 C92 C93 C94 C95 | 2 |
| Lymphoma | C81 C82 C83 C84 C85 C88 C90 C96 | 2 |
| Moderate/severe liver disease | B150 B160 B162 B190 K704 K72 K766 I85 | 3 |
| Metastatic solid tumor | C76 C77 C78 C79 C80 | 6 |
| AIDS | B21 B22 B23 B24 | 6 |

| **CHA_2_DS_2_-VASc indicator** | **ICD-10 or ATC codes** | **Weight** |
| --- | --- | --- |
| **Congestive Heart failure** | **ICD-10 codes:** I110, I130, I132, I420, I50, C03C | 1 |
| **Hypertension** | **ICD-10 codes:** I10 I11 I12 I13 I15  **ATC codes:** C02A C02B C02C C02DA C02L C03A C03B C03D C03EA C03X C07C C07D C08G C09BA C09DA C09XA52 C02DB C02DD C02DG C04 C05 C07 C07F C08 C09BB C09DB C09  C09BB04 C09DA C09DB C09CX01 C09DX04 C07B | 1 |
| **Age ≥75** |  | 1 |
| **Age ≥65** |  | 1 |
| **Diabetes** | **ICD-10 codes:** E10, E11, E12, E13, E14  **ATC codes:** A10 | 1 |
| **Stroke** | **ICD-10 codes:** I63, I64, I74, G45 | 2 |
| **Vascular Disease** | **ICD-10 codes:** I21, I23, I702, I708, I709, I71, I739, I700 | 1 |
| **Female Sex** |  | 1 |

**Supplementary Table 2. Sensitivity analysis MACE Outcomes by Cancer Type within 1 year of follow-up**

|  | **MACE, main analysis** | | | **MACE, MACE free at entry subgroup** | | |
| --- | --- | --- | --- | --- | --- | --- |
| **Cancer strata** | **Events** | **Event Rate**  **pr 100 PY (CI)** | **1-year cumulative incidence % (CI)** | **Events** | **Event Rate**  **pr 100 PY (CI)** | **1-year cumulative incidence % (CI)** |
| All | 2,517 | 3.59 (3.45-3.73) | 2.8 (2.7-2.9) | 1,187 | 2.79 (2.63-2.95) | 2.2 (2.1-2.3) |
| Digestive tract | 681 | 3.96 (3.67-4.26) | 2.8 (2.6-3.0) | 310 | 3.01 (2.69-3.37) | 2.2 (2.0-2.5) |
| Respiratory | 556 | 4.82 (4.44-5.24) | 2.9 (2.7-3.2) | 283 | 4.05 (3.61-4.55) | 2.6 (2.3-2.9) |
| Bone, Skin, Tissue | 100 | 2.34 (1.92-2.85) | 2.1 (1.7-2.5) | 45 | 1.72 (1.28-2.30) | 1.6 (1.2-2.1) |
| Glandular, reproductive | 648 | 2.71 (2.51-2.92) | 2.5 (2.3-2.7) | 299 | 1.99 (1.78-2.23) | 1.9 (1.7-2.1) |
| Uro-renal | 245 | 4.18 (3.69-4.73) | 3.3 (2.9-3.8) | 115 | 3.45 (2.87-4.14) | 2.8 (2.3-3.3) |
| Neurologic | 35 | 4.07 (2.92-5.67) | 2.4 (1.7-3.3) | 15 | 3.12 (1.88-5.17) | 1.9 (1.1-3.1) |
| Hematologic | 252 | 3.95 (3.49-4.47) | 3.1 (2.8-3.5) | 120 | 3.09 (2.59-3.70) | 2.5 (2.1-3.0) |
|  | **MACE, acute, elective, primary, secondary outcome** | | |  | | |
| All | 4,557 | 6.60 (6.41-6.79) | 5.0 (4.9-5.2) |  |  |  |
| Digestive tract | 1,318 | 7.80 (7.39-8.24) | 5.4 (5.2-5.7) |  |  |  |
| Respiratory | 980 | 8.63 (8.11-9.19) | 5.2 (4.9-5.5) |  |  |  |
| Bone, Skin, Tissue | 181 | 4.29 (3.70-4.96) | 3.8 (3.3-4.4) |  |  |  |
| Glandular, reproductive | 1,105 | 4.67 (4.40-4.95) | 4.2 (4.0-4.5) |  |  |  |
| Uro-renal | 432 | 7.48 (6.81-8.22) | 5.9 (5.4-6.4) |  |  |  |
| Neurologic | 93 | 11.09 (9.05-13.59) | 6.4 (5.2-7.7) |  |  |  |
| Hematologic | 448 | 7.12 (6.49-7.81) | 5.5 (5.1-6.0) |  |  |  |

*MACE includes ischemic stroke, systemic embolism, myocardial infarction, and hemorrhagic stroke. Only acute, primary diagnoses were included, unless stated otherwise. CI: Confidence interval (95%)*

**Supplementary Table 3. Sensitivity analysis VTE Outcomes by Cancer Type within 1 year of follow-up**

|  | **VTE, main analysis** | | | **VTE, VTE free at entry subgroup** | | |
| --- | --- | --- | --- | --- | --- | --- |
| **Cancer strata** | **Events** | **Event Rate**  **pr 100 PY (CI)** | **1-year cumulative incidence % (CI)** | **Events** | **Event Rate**  **pr 100 PY (CI)** | **1-year cumulative incidence % (CI)** |
| All | 1,658 | 2.36 (2.25-2.47) | 1.8 (1.7-1.9) | 1,323 | 2.00 (1.90-2.11) | 1.6 (1.5-1.6) |
| Digestive tract | 551 | 3.19 (2.94-3.47) | 2.3 (2.1-2.5) | 439 | 2.72 (2.48-2.99) | 2.0 (1.8-2.1) |
| Respiratory | 449 | 3.88 (3.54-4.26) | 2.4 (2.2-2.6) | 355 | 3.28 (2.95-3.63) | 2.0 (1.8-2.2) |
| Bone, Skin, Tissue | 40 | 0.93 (0.68-1.27) | 0.8 (0.6-1.1) | 33 | 0.82 (0.58-1.15) | 0.7 (0.5-1.0) |
| Glandular, reproductive | 304 | 1.26 (1.13-1.41) | 1.2 (1.0-1.3) | 240 | 1.06 (0.93-1.20) | 1.0 (0.9-1.1) |
| Uro-renal | 121 | 2.04 (1.71-2.44) | 1.6 (1.4-2.0) | 104 | 1.88 (1.55-2.27) | 1.5 (1.3-1.8) |
| Neurologic | 45 | 5.26 (3.93-7.05) | 3.1 (2.3-4.1) | 35 | 4.34 (3.12-6.05) | 2.6 (1.8-3.5) |
| Hematologic | 148 | 2.31 (1.97-2.72) | 1.8 (1.6-2.1) | 117 | 1.96 (1.64-2.35) | 1.6 (1.3-1.9) |
|  | **VTE, acute, elective, primary, secondary outcome** | | | **VTE, up to 20 days to confirmation** | | |
| All | 2,432 | 3.48 (3.34-3.62) | 2.7 (2.6-2.8) | 1,829 | 2.60 (2.49-2.72) | 2.0 (1.9–2.1) |
| Digestive tract | 804 | 4.69 (4.38-5.03) | 3.3 (3.1-3.5) | 621 | 3.61 (3.33–3.90) | 2.6 (2.4–2.8) |
| Respiratory | 614 | 5.34 (4.93-5.77) | 3.2 (3.0-3.5) | 495 | 4.29 (3.92–4.68) | 2.6 (2.4–2.9) |
| Bone, Skin, Tissue | 66 | 1.54 (1.21-1.96) | 1.4 (1.1-1.7) | 45 | 1.05 (0.78–1.41) | 0.9 (0.7–1.2) |
| Glandular, reproductive | 453 | 1.89 (1.72-2.07) | 1.7 (1.6-1.9) | 326 | 1.36 (1.22–1.51) | 1.3 (1.1–1.4) |
| Uro-renal | 192 | 3.27 (2.84-3.76) | 2.6 (2.3-3.0) | 137 | 2.32 (1.96–2.74) | 1.9 (1.6–2.2) |
| Neurologic | 57 | 6.71 (5.17-8.70) | 3.9 (3.0-5.0) | 45 | 5.26 (3.93–7.05) | 3.1 (2.3–4.1) |
| Hematologic | 246 | 3.88 (3.42-4.39) | 3.0 (2.7-3.4) | 160 | 2.50 (2.14–2.92) | 2.0 (1.7–2.3) |

*VTE includes pulmonary embolism and deep vein thrombosis, confirmed by diagnostic evaluation (imaging or ultra-sound). Only acute, primary diagnoses were included, unless stated otherwise. CI: Confidence interval (95%)*

**Supplementary Table 4. Hazard ratios (HR) for cardiovascular death, MACE, and VTE across clinical variables and cancer**

| **Exposure** | **Cardiovascular death** | | **MACE** | | **VTE** | |
| --- | --- | --- | --- | --- | --- | --- |
|  | **Crude** | **Adjusted** | **Crude** | **Adjusted** | **Crude** | **Adjusted** |
|  | **HR (95% CI)** | **HR (95% CI)** | **HR (95% CI)** | **HR (95% CI)** | **HR (95% CI)** | **HR (95% CI)** |
| **Female** | **0.87 (0.80–0.95)** | **0.81 (0.74–0.89)** | **0.84 (0.77–0.91)** | **0.86 (0.79–0.94)** | 1.05 (0.95–1.16) | 1.03 (0.93–1.15) |
| **Cancer group** |  |  |  |  |  |  |
| Respiratory | **1.63 (1.29–2.05)** | **1.75 (1.39–2.22)** | **1.94 (1.57–2.40)** | **1.86 (1.50–2.30)** | **3.89 (2.81–5.37)** | **3.89 (2.81–5.38)** |
| Bone, Skin, Tissue | Reference | Reference | Reference | Reference | Reference | Reference |
| Glandular, reproductive | 1.10 (0.87–1.38) | 1.17 (0.94–1.48) | 1.16 (0.94–1.43) | 1.19 (0.96–1.47) | 1.36 (0.98–1.90) | 1.37 (0.98–1.90) |
| Digestive tract | **1.79 (1.43–2.25)** | **1.74 (1.39–2.18)** | **1.64 (1.33–2.02)** | **1.57 (1.27–1.93)** | **3.30 (2.40–4.55)** | **3.37 (2.44–4.64)** |
| Hematologic | **1.96 (1.53–2.50)** | **1.94 (1.52–2.47)** | **1.66 (1.32–2.09)** | **1.62 (1.29–2.04)** | **2.44 (1.72–3.46)** | **2.43 (1.71–3.44)** |
| Neurologic | **1.70 (1.14–2.54)** | **2.10 (1.40–3.13)** | **1.61 (1.09–2.26)** | **1.68 (1.14–2.46)** | **5.15 (3.36–7.88)** | **4.99 (3.26–7.65)** |
| Uro-renal | **1.87 (1.46–2.40)** | **1.74 (1.36–2.23)** | **1.75 (1.39–2.21)** | **1.61 (1.27–2.03)** | **2.15 (1.50–3.07)** | **2.19 (1.53–3.13)** |
| **Charlson group** |  |  |  |  |  |  |
| 4 | **2.45 (2.17–2.76)** | **2.07 (1.83–2.34)** | **1.59 (1.43–1.76)** | **1.45 (1.31–1.61)** | 1.01 (0.90–1.15) | 0.95 (0.84–1.08) |
| 5 | **3.45 (3.03–3.93)** | **2.72 (2.39–3.11)** | **1.77 (1.56–1.99)** | **1.56 (1.38–1.76)** | 1.03 (0.89–1.20) | 0.96 (0.82–1.12) |
| 6 | **4.20 (3.64–4.84)** | **3.37 (2.92–3.90)** | **2.16 (1.89–2.48)** | **1.92 (1.67–2.21)** | 1.07 (0.89–1.29) | 0.98 (0.81–1.19) |
| 7 | **4.61 (4.05–5.25)** | **3.84 (3.35–4.39)** | **2.30 (2.04–2.60)** | **2.05 (1.80–2.32)** | 0.87 (0.73–1.05) | **0.77 (0.64–0.94)** |
| Atrial fibrillation | **2.33 (2.14–2.54)** | **1.48 (1.32–1.65)** | **1.25 (1.14–1.37)** | 1.12 (0.99–1.26) | **0.63 (0.55–0.73)** | **0.70 (0.59–0.83)** |
| Previous bleeding | **1.72 (1.56–1.88)** | **1.19 (1.08–1.31)** | **1.35 (1.23–1.48)** | **1.15 (1.05–1.27)** | **0.84 (0.74–0.96)** | **0.79 (0.69–0.91)** |
| Heart failure | **3.34 (3.07–3.65)** | **2.29 (2.08–2.53)** | **1.51 (1.39–1.63)** | **1.22 (1.12–1.33)** | 0.91 (0.82–1.00) | 0.97 (0.86–1.08) |
| Myocardial infarction | **1.57 (1.44–1.71)** | **1.51 (1.38–1.65)** | **1.43 (1.32–1.55)** | **1.47 (1.35–1.60)** | 0.96 (0.86–1.06) | 1.00 (0.89–1.12) |
| Peripheral artery disease | **1.42 (1.30–1.55)** | **1.43 (1.30–1.56)** | **1.40 (1.29–1.52)** | **1.38 (1.27–1.51)** | 1.10 (0.99–1.22) | 0.93 (0.83–1.03) |
| Stroke | **2.14 (1.96–2.34)** | **1.64 (1.49–1.80)** | **1.74 (1.59–1.90)** | **1.51 (1.38–1.66)** | 1.04 (0.91–1.17) | 1.03 (0.90–1.17) |
| Venous thromboembolism | **1.30 (1.11–1.51)** | 1.01 (0.87–1.18) | **1.24 (1.07–1.44)** | 1.13 (0.97–1.31) | **3.82 (3.39–4.31)** | **4.06 (3.58–4.62)** |
| Thyroid disease | 1.16 (1.00-1.35) | 1.04 (0.89–1.21) | 0.91 (0.78-1.06) | 0.91 (0.78–1.07) | 0.94 (0.78-1.14) | 0.94 (0.77–1.14) |
| Rheumatoid arthritis | 1.03 (0.91–1.17) | 0.86 (0.75–0.97) | 1.07 (0.95–1.20) | 0.98 (0.88–1.11) | 1.03 (0.89–1.19) | 0.98 (0.84–1.13) |
| Renal disease | **2.24 (1.99–2.52)** | **1.42 (1.26–1.61)** | **1.38 (1.21–1.58)** | 1.07 (0.93–1.23) | 0.97 (0.80–1.17) | 1.00 (0.82–1.22) |
| Liver disease | 0.88 (0.66–1.18) | 0.96 (0.71–1.28) | 0.79 (0.59–1.05) | 0.75 (0.56–1.01) | 1.21 (0.91–1.62) | 0.95 (0.71–1.28) |
| Diabetes | **1.28 (1.16–1.41)** | 1.06 (0.96–1.17) | **1.30 (1.19–1.42)** | **1.19 (1.08–1.31)** | 0.97 (0.87–1.10) | 0.99 (0.87–1.12) |
| Hypertension | **1.84 (1.61–2.10)** | 0.92 (0.79–1.07) | **1.35 (1.21–1.51)** | 1.08 (0.96–1.22) | 0.82 (0.73–0.93) | 0.94 (0.83–1.07) |
| Statin treatment | **0.74 (0.68–0.80)** | **0.65 (0.59–0.71)** | 1.01 (0.93–1.10) | **0.83 (0.76–0.91)** | 0.92 (0.83–1.02) | 0.99 (0.89–1.11) |
| Oral anticoagulation | **1.78 (1.62–1.96)** | 0.96 (0.85–1.09) | **1.12 (1.01–1.24)** | 0.88 (0.77–1.01) | 0.88 (0.77–1.01) | 0.85 (0.71–1.01) |
| Antiplatelet treatment | **1.18 (1.08–1.30)** | **1.22 (1.10–1.35)** | **1.29 (1.18–1.41)** | **1.15 (1.04–1.27)** | **0.89 (0.80–0.98)** | **0.88 (0.78–0.99)** |

*Values in bold represent statistically significant associations for the adjusted estimates (95% CI excludes 1.00).
MACE includes ischemic stroke, systemic embolism, myocardial infarction, and hemorrhagic stroke.* *VTE includes pulmonary embolism and deep vein thrombosis, confirmed by diagnostic evaluation (imaging or ultra-sound). Only acute, primary diagnoses were included. Cardiovascular death was identified using the Danish Cause of Death Register. CI: Confidence interval (95%).*

**Supplementary Table 5. Fine-Gray sub-distribution Hazard ratios (sHR) for cardiovascular death, MACE, and VTE across clinical variables and cancer**

| **Exposure** | **Cardiovascular death** | | **MACE** | | **VTE** | |
| --- | --- | --- | --- | --- | --- | --- |
|  | **Crude** | **Adjusted** | **Crude** | **Adjusted** | **Crude** | **Adjusted** |
|  | **sHR (95% CI)** | **sHR (95% CI)** | **sHR (95% CI)** | **sHR (95% CI)** | **sHR (95% CI)** | **sHR (95% CI)** |
| **Female** | **0.86 (0.79–0.94)** | **0.81 (0.74–0.89)** | **0.83 (0.76–0.90)** | **0.86 (0.79–0.94)** | 1.03 (0.93–1.14) | 1.03 (0.93–1.15) |
| **Cancer group** |  |  |  |  |  |  |
| Respiratory | 1.22 (0.97–1.54) | 1.20 (0.95–1.51) | **1.41 (1.14–1.75)** | **1.32 (1.06–1.63)** | **2.86 (2.07–3.95)** | **2.88 (2.08–3.99)** |
| Bone, Skin, Tissue | Reference | Reference | Reference | Reference | Reference | Reference |
| Glandular, reproductive | 1.12 (0.89–1.41) | 1.18 (0.94–1.48) | 1.19 (0.96–1.47) | 1.21 (0.98–1.49) | 1.39 (1.00–1.94) | 1.40 (1.00–1.94) |
| Digestive tract | **1.51 (1.21–1.90)** | **1.38 (1.11–1.73)** | **1.35 (1.10–1.67)** | **1.28 (1.04–1.58)** | **2.74 (1.99–3.78)** | **2.87 (2.08–3.96)** |
| Hematologic | **1.79 (1.40–2.28)** | **1.68 (1.32–2.15)** | **1.50 (1.19–1.89)** | **1.45 (1.15–1.82)** | **2.20 (1.55–3.11)** | **2.23 (1.57–3.16)** |
| Neurologic | 1.26 (0.85–1.89) | 1.33 (0.89–1.98) | 1.15 (0.79–1.70) | 1.13 (0.77–1.66) | **3.74 (2.44–5.72)** | **3.72 (2.43–5.70)** |
| Uro-renal | **1.74 (1.36–2.23)** | **1.55 (1.21–1.98)** | **1.61 (1.27–2.03)** | **1.47 (1.17–1.86)** | **1.98 (1.38–2.83)** | **2.05 (1.43–2.93)** |
| **Charlson group** |  |  |  |  |  |  |
| 4 | **2.29 (2.02–2.58)** | **1.99 (1.76–2.25)** | **1.47 (1.32–1.63)** | **1.38 (1.24–1.53)** | 0.94 (0.83–1.06) | 0.90 (0.80–1.02) |
| 5 | **3.09 (2.72–3.52)** | **2.53 (2.21–2.89)** | **1.55 (1.37–1.75)** | **1.43 (1.26–1.61)** | 0.91 (0.78–1.06) | 0.87 (0.75–1.02) |
| 6 | **3.69 (3.21–4.26)** | **3.02 (2.61–3.50)** | **1.86 (1.62–2.13)** | **1.70 (1.48–1.95)** | 0.92 (0.76–1.11) | 0.87 (0.72–1.05) |
| 7 | **3.89 (3.41–4.42)** | **3.29 (2.87–3.77)** | **1.88 (1.66–2.12)** | **1.72 (1.51–1.95)** | **0.72 (0.60–0.86)** | **0.64 (0.53–0.78)** |
| Atrial fibrillation | **2.20 (2.02–2.40)** | **1.42 (1.27–1.59)** | **1.16 (1.06–1.27)** | 1.07 (0.95–1.20) | **0.59 (0.51–0.68)** | **0.67 (0.56–0.81)** |
| Previous bleeding | **1.62 (1.48–1.78)** | **1.18 (1.07–1.30)** | **1.26 (1.15–1.38)** | **1.14 (1.04–1.25)** | **0.79 (0.69–0.90)** | **0.78 (0.68–0.89)** |
| Heart failure | **3.10 (2.84–3.38)** | **2.22 (2.00–2.45)** | **1.37 (1.26–1.48)** | **1.16 (1.06–1.27)** | 0.82 (0.74–0.91) | 0.92 (0.82–1.03) |
| Myocardial infarction | **1.56 (1.43–1.70)** | **1.49 (1.35–1.63)** | **1.42 (1.31–1.53)** | **1.43 (1.31–1.57)** | 0.94 (0.85–1.05) | 0.98 (0.87–1.10) |
| Peripheral artery disease | **1.33 (1.22–1.45)** | **1.39 (1.27–1.52)** | **1.30 (1.20–1.41)** | **1.33 (1.22–1.45)** | 1.02 (0.92–1.13) | 0.89 (0.80–1.00) |
| Stroke | **2.00 (1.83–2.19)** | **1.58 (1.44–1.74)** | **1.60 (1.46–1.75)** | **1.44 (1.32–1.58)** | 0.95 (0.84–1.08) | 0.97 (0.86–1.11) |
| Venous thromboembolism | **1.21 (1.04–1.42)** | 0.96 (0.82–1.12) | 1.15 (0.99–1.33) | 1.08 (0.93–1.26) | **3.55 (3.15–4.00)** | **3.89 (3.43–4.41)** |
| Thyroid disease | 1.16 (1.00–1.34) | 1.05 (0.90–1.22) | 0.90 (0.77–1.05) | 0.92 (0.79–1.08) | 0.94 (0.77–1.13) | 0.96 (0.79–1.16) |
| Rheumatoid arthritis | 1.02 (0.90–1.16) | 0.87 (0.76–0.98) | 1.06 (0.94–1.19) | 1.00 (0.89–1.13) | 1.02 (0.89–1.18) | 0.99 (0.86–1.15) |
| Renal disease | **2.06 (1.83–2.32)** | **1.32 (1.16–1.50)** | **1.25 (1.09–1.42)** | 1.00 (0.87–1.15) | 0.87 (0.72–1.06) | 0.95 (0.78–1.16) |
| Liver disease | 0.79 (0.59–1.06) | 0.88 (0.65–1.18) | **0.70 (0.53–0.94)** | **0.85 (0.51–0.92)** | 1.08 (0.81–1.45) | 0.86 (0.64–1.17) |
| Diabetes | **1.23 (1.12–1.35)** | 1.02 (0.92–1.12) | **1.24 (1.13–1.35)** | **1.14 (1.04–1.26)** | 0.93 (0.83–1.05) | 0.94 (0.83–1.07) |
| Hypertension | **1.81 (1.59–2.08)** | 0.95 (0.82–1.10) | **1.33 (1.19–1.49)** | 1.11 (0.98–1.26) | **0.81 (0.72–0.91)** | 0.96 (0.85–1.10) |
| Statin treatment | **0.77 (0.71–0.84)** | **0.69 (0.63–0.76)** | 1.07 (0.99–1.16) | **0.89 (0.81–0.98)** | 0.97 (0.88–1.07) | 1.06 (0.94–1.18) |
| Oral anticoagulation | **1.73 (1.57–1.90)** | 1.00 (0.88–1.14) | 1.07 (0.97–1.19) | 0.92 (0.80–1.05) | **0.84 (0.73–0.97)** | 0.86 (0.71–1.03) |
| Antiplatelet treatment | **1.18 (1.08–1.29)** | **1.22 (1.09–1.35)** | **1.28 (1.18–1.40)** | **1.15 (1.04–1.27)** | **0.88 (0.80–0.98)** | **0.87 (0.77–0.98)** |

*Values in bold represent statistically significant associations for the adjusted estimates (95% CI excludes 1.00).
MACE includes ischemic stroke, systemic embolism, myocardial infarction, and hemorrhagic stroke. VTE includes pulmonary embolism and deep vein thrombosis, confirmed by diagnostic evaluation (imaging or ultra-sound). Only acute, primary diagnoses were included. Cardiovascular death was identified using the Danish Cause of Death Register. CI: Confidence interval (95%).*

**Supplementary Figure 1. Mortality up to one year of follow-up by cancer type.**

**Supplementary Figure 2. Risk Factors for Cardiovascular Death, MACE and VTE After a Cancer Diagnosis**

*Forest plots of subdistribution hazard ratios (sHRs) and 95% confidence intervals for cardiovascular death, MACE, and VTE. Both crude (blue) and adjusted (red) estimates are shown for each risk factor. MACE includes ischemic stroke, systemic embolism, myocardial infarction, and hemorrhagic stroke. VTE includes pulmonary embolism and deep vein thrombosis.*
